# Perceptions of nurses on TB with HIV adherence counselling skills in a health sub-district, Cape Town: A qualitative study

**DOI:** 10.4102/hsag.v27i0.1859

**Published:** 2022-07-26

**Authors:** Victoire Ticha, Million Bimerew, Deliwe R. Phetlhu

**Affiliations:** 1 Department of Nursing, Faculty of Community and Health Sciences, University of the Western Cape, Cape Town, South Africa; 2 Department of Nursing, Faculty of Health Sciences, Sefako Makgatho Health Sciences University, Pretoria, South Africa

**Keywords:** HIV, TB, co-infection, dual stigma, adherence counselling, education, nurse

## Abstract

**Background:**

People living with HIV (PLHIV) co-infected with Tuberculosis (TB) account for one in three HIV-related deaths. Retention in care and adherence to medication remain key behaviours that PLHIV co-infected with TB must adopt to achieve better health outcomes. Nevertheless, TB with HIV adherence-counselling services provided by nurses designed to enhance these behaviours remain inadequate. Additionally, limited information is found in the literature on the perceptions of nurses regarding their TB with HIV adherence counselling skills pertaining to PLHIV co-infected with TB.

**Aim:**

To explore and describe the perceptions of nurses regarding their TB with HIV adherence counselling skills of PLHIV co-infected with TB.

**Setting:**

The study was conducted in a health sub-district of Cape Town.

**Method:**

An exploratory, descriptive qualitative design was followed. A total of 14 purposively sampled nurses were interviewed individually. Nurses caring for PLHIV co-infected with TB were included and nurses not offering care to PLHIV co-infected with TB were excluded. All interviews were audio recorded with the participants’ permission followed by verbatim transcriptions. Thematic analysis was done using ATLASti.8 electronic software.

**Results:**

It was established that the varied roles of these nurses increased their workload. Nonetheless, despite the gap in their counselling skills, the majority still maintained work expertise, professionalism and empathy towards the patients. Additionally, there were perceived barriers impacting patients’ attendance of their follow up appointments.

**Conclusion:**

Based on the findings of this study, there is a need to equip nurses caring for PLHIV co-infected with TB with adherence counselling skills to improve practice.

**Contribution:**

The findings were synthesised with results from an in-depth literature review to stand as the backbone for the development of a training programme for nurses to improve adherence counselling.

## Introduction

In 2017 an estimated 11% of patients infected with Mycobacterium Tuberculosis were co-infected with HIV (Joint United Nations Protocol on HIV/AIDS [UNAIDS] 2017). Tuberculosis is also the leading cause of death among PLHIV, accounting for one in three HIV‑related deaths (UNAIDS 2016; WHO 2018). Worldwide, TB is one of the top 10 causes of death and the leading cause from a single infectious agent (WHO 2020b). Around one third of the 36.9 million PLHIV and AIDS worldwide are co-infected with TB (WHO 2020). Sub-Saharan Africa is the hardest hit region, with 70% of all people living with HIV co-infected with TB in the world (UNAIDS 2016). The Stop TB Partnership and the Global Fund to Fight AIDS, Tuberculosis and Malaria launched Find, Treat All, a joint initiative to scale up the End TB response towards universal access to TB prevention and care (WHO 2020). This initiative stresses the need for a multisectoral approach to addressing the specific needs of people living with HIV with TB co-infection (WHO 2018). A total of 1.4 million people died from TB in 2019 (including 208 000 people with HIV).

According to the South Africa Strategy Plan (NSP) on HIV, TB and STIs (SANAC n.d.), an estimated 270 000 people became newly diagnosed with HIV and the 2015 estimate of new TB cases was 450 000. An estimated 64 000 total deaths due to TB infection were reported in 2019 in South Africa (WHO 2019). The Western Cape province in 2016 recorded 74.6% of PLHIV co-infected with TB (Massyn, Pillay & Padarath 2019) with the Cape Town district recording 81.1% of the total co-infections of HIV with TB patients on ART in the province (Massyn et al. 2018). According to the WHO Global Tuberculosis Report: South Africa in 2018, 104 625 HIV with TB cases were recorded (WHO 2019). According to UNAIDS, in 2019, less than half (49%) of the estimated 815 000 people living with HIV who also have TB disease were reported to be receiving both HIV treatment and TB treatment (UNAIDS 2020).

Adherence to concurrent TB treatment and ART amongst co-infected persons is a major barrier to successful treatment outcomes (Mazinyo et al. 2016). The burdens of taking tablets, length of treatment, maintaining secrecy of HIV with TB condition, patient–provider relationship, patient–household interaction, alcohol intake and stigma still remain inevitable challenges faced by people living with HIV co-infected with TB (Mandimika & Friedland 2020; Mbunyuza 2020). Most PLHIV co-infected with TB have identified treatment fatigue related to pill burden as a major barrier to their medication adherence (Mazinyo et al. 2016). To overcome medication non-adherence among these patients, proper adherence counselling is required (Ndwiga, Kikuvi & Omolo 2016). It is required that such counselling should be conducted by an appropriately trained, mentored and supervised counsellor or healthcare worker (South Africa Department of Health 2015). Although continuous adherence counselling is identified as important to enhance medication adherence (Southern African HIV Clinicians Society n.d.), the number of quality healthcare personnel to offer proper counselling remains a barrier (Mahtab & Coetzee 2017). HIV with TB adherence can be further aggravated by having insufficient disease knowledge, thus adherence counselling services by nurses can bridge such a knowledge gap. Already the clock is ticking for us to reach the ambitious new 2025 targets for TB and HIV laid out in the new Global AIDS Strategy for 2021–2026 (End Inequalities. End AIDS. Global AIDS Strategy 2021–2026). Adherence counselling by all the cadre of nurses will facilitate reaching the target. Therefore, the aim of this study was to explore and describe the perceptions of nurses regarding the adherence-counselling skills of PLHIV co-infected with TB in a health sub-district of Cape Town.

Attempts have been made at scientific and policy levels to improve the health outcomes of PLHIV co-infected with TB, including improving the adherence-counselling services offered to them. One such policy is shifting the task of patient adherence counselling to nurses and lay counsellors or community health workers. Task shifting roles of both nurses and lay counselors should be clearly defined because the lay counselors are responsible for different counseling tasks at the Community Health Care Centers (CHCs). Although task-shifting is practised in some CHCs between the adherence counsellors and the nurses, the nurses are not trained in adherence counselling as the adherence counsellors are (Fernando & Msf 2016).

Phetlhu et al. (2018) acknowledged that most of the nurses have insufficient knowledge and skill to provide TB and HIV-integrated care on certain aspects stipulated in the treatment guidelines such as medications, counselling, intensive case findings and management of multidrug-resistant TB. Also, nurses who had been working as TB nurses identified the need to become counsellors considering the increasing number of TB patients that required HIV counselling (Njozing et al. 2011).

TB with HIV counselling skills remain critically inadequate as this has been identified by nurses with the integrated treatment of PLHIV co-infected with TB. TB with HIV adherence-counselling services provided by nurses designed to enhance patient compliance remain inadequate.

**Study objective:** To explore and describe the perceptions of the nurses regarding TB with HIV adherence counselling skills in a health sub-district in Cape Town.

### Research methods and design

An exploratory, descriptive, qualitative research design was employed in this study (Burns & Grove 2009). This design was adopted to guide the researchers to explore and describe the perspectives of the nurses offering adherence counselling services to PLHIV co-infected with TB. Duration was from October 2020 to June 2021.

### Study setting

The Cape Town metropole consists of eight health sub-districts, namely, Tygerberg, Khayelitsha, Helderberg or Eastern, Northern Panorama, Central or Western, Southern, Klipfontein and Mitchells Plain Western Cape Department of Health (Western Cape Government n.d.). Khayelitsha has nine clinics and for this study, five clinics which offer Voluntary Counseling and Testing (VCT), Prevention of Mother To Child Transmission (PMTCT), ART and TB screening and management were included. These five facilities were purposively selected because they service the highest number of PLHIV and TB in this sub-district based on head count, and these facilities also record the highest numbers of loss-to-follow-up of PLHIV co-infected with TB (Massyn, Pillay & Padarath 2017). These factors provide the opportunity to explore and describe the perceptions of nurses regarding adherence counselling skills of PLHIV co-infected with TB in a health sub-district of Cape Town.

### Participant recruitment

There are approximately 180 nurses in the nine CHCs in the Khayelitsha health sub-district (SAMU 2014) and 80 nurses working in the five selected CHCs. All nurses working in the selected CHCs (80) and offering services to PLHIV co-infected with TB formed the population of the study. Nurses who do not offer TB with HIV related services were excluded. Fourteen nurses participated in this study selected from two clinics, based on their willingness to take part in the study.

### Sampling technique

Purposive sampling was used to recruit the sample of nurses who participated in the study. The decision was based on the day-to-day interactions and experience of these nurses caring for PLHIV co-infected with TB at the selected CHCs. Thus they were able to provide information required for this study. Sample size was not calculated because of the small population size.

### Preparation of participants

Participants were selected if (1) they were registered with the South African Nursing Council (SANC) and (2) they offered care to PLHIV co-infected with TB at the health sub-district. After doing a PowerPoint presentation to the nurses, they were requested to volunteer to participate in the study interviews. The purpose of the study was explained to the participants, and informed consent was obtained before the interview began. Dates, time and venue were arranged, and the majority of the participants preferred the staff tea rooms for the interviews whilst others used their consultation rooms.

### Data collection

Data were collected using semi-structured interviews in English. Open-ended questions were used to start each session and probes were used to encourage active participation of the participants and for clarity. This approach allowed the participants to respond in their own words and it offered flexibility (Brink, Van der Walt & Van Rensburg 2012). The question posed was as follows: what is your perception about your skills to conduct adherence counselling to PLHIV co-infected with TB? During the interview sessions, the majority of the participants felt at ease to express themselves. The use of gestures and body language were also observed. Interviews were recorded using an audio recorder with permission from the study participants. Field notes were also taken during each interview session. The interview sessions lasted between 45 and 60 min. A code CHC101 was allocated to each interview with the first letters representing the CHC and the last three numbers representing the participant. At the end of data collection, the participants were thanked.

### Data analysis

Data analysis occurred concurrently with data collection (Burns & Grove 2010). Data were imported into ATLAS.ti 8 software and analysed thematically. ATLAS.ti 8 was used for organising the transcripts and audio files to facilitate coding (Creswell 2009). Thematic analysis was employed to conduct the analysis. Initial coding was done by slowly reading through each interview transcript to identify sections of the text that respond to the research questions. The codes were grouped into code families and then reviewed to identify emerging themes. Some codes were attached to the comments to further elaborate the content of the code (Creswell 2016). An independent coder, experienced in the field of qualitative research and HIV research, independently coded a copied set of the raw data. Meetings were held between the researchers and the independent coder to compare and verify themes and categories that had emerged between the two analyses.

### Ethical considerations

Ethical clearance was obtained from the Biomedical Research Ethics Committee of the University of the Western Cape (No. BM19/8/9). The research also obtained approval from the Western Cape Department of Health (No. WC_201910_036) and City of Cape Town (No. 8230).

Informed consent was obtained from participants via a consent form which included permission to audio record. This study, confidentiality was maintained by ensuring that data were not linked to any participant’s name. Voice recordings and data in soft copy versions were kept strictly confidential on a laptop encrypted with a password known only to the research team. Data anonymity was maintained by the use of codes. There was no foreseeable physical risk in this study.

## Results

Fourteen participants took part in the study, consisting of 10 female and 4 male nurses. Extracts from the participants were used to support the descriptions of the themes. The exact language and phrases used by the participants were maintained, but for the sake of clarity, some grammatical amendments were made. Table 1 provides a summary of the emerged themes and categories.

**TABLE 1:** Emerged themes and categories.

Themes	Categories
The complexity of the varied roles of the nurses	Educational role of nurses Perceived providing adherence counselling as one of the function of nurses. Referral role as function of the nurses
Display of professionalism by these nurses despite challenges	Importance of positive relationships (encouragement and reassurance)
The role of expertise and experience	Counselling from experience
Perceived patient barriers to attend CHC follow-up appointments impacting on nurses’ role	Employer-related barriers Community-related barriers Substance use

CHC, community health care center.

### Theme 1: The complexity of the varied roles of the nurses

The nurses collaborative roles with external staff and lay counselors improved their adherence counseling skills. This resulted in the first theme which reflected the complexity of the varied roles of the nurses. This theme had three categories.

#### Category 1.1: Educational role of nurses

Participants reported that they performed many roles embedded in the adherence counselling of PLHIV co-infected with TB. The educational role was one of the key roles.

‘Then, when you’re starting the treatment, you explain why it is important to take treatment and you explain to the patient that this is a lifelong treatment; “you are not going to stop the treatment.” You just browse and tell that each and every treatment is different, most of the treatment do cause side effects.’ (Participant CHC 101, female, Enrolled Nurse (EN))

However, there were some challenges experienced while carrying out counselling of this patients, especially time constraints and workload.

‘This clinic is too big to do ARVs and TB. You don’t have time to reflect and because the folder is like this [indicates a huge volume], and they’re always sitting there and they are talking, no time. So it’s only when then you miss most of the things, because what you’re doing is just to give them tablets.’ (Participant CHC301, male, Professional Nurse (PN))

Also, the educational role of nurses also served as a source of acquiring skills to carry out adherence counselling for PLHIV co-infected with TB because of their close interactions with the patients (human relationships), hence a source of equipping the nurses with adherence counselling skills.

‘Because, with most of the time you will find out that they are not taking the treatment; especially the TB ones, because they believe that if they finish the 6 months they’re not going to get the disability grant again. As soon as they get the grant, they stop the treatment and they manipulate the system. So you try even then to educate them it’s not about money; it’s about your health. When not taking the treatment, your body will be a little resistant and when you die the money will be here.’ (Participant CHC301, male, PN)

Although adherence counselling through education is noted, participant CHC101 had this to say:

‘I think I don’t have so much skills in adherence counselling because we don’t normally do the adherence counselling. The adherence counselling is done by our counsellors.’ (Participant CHC101, female, EN)

#### Category 1.2: Perceived providing adherence counselling as one of the function of nurses

Providing adherence counselling services, although important, was not currently one of the key roles of the nurses. Below is what the participants reported:

‘I don’t have so much skills in adherence counselling because we don’t normally do the adherence counselling. The adherence counselling is done by our counsellors.’ (Participant CHC303, male, PN)

‘I seldom do adherence counselling; it is very important for us to do adherence counselling, yes. From the nurses there in the front, adherence counselling needs to be done all the way. It is very important, so that patients can adhere to the treatment. And they need to know the importance, the benefits and all. So it is very important.’ (Participant CHC103, female, PN)

According to participant 10, a RN from the fourth CHC, counsellors are better trained to offer adherence counselling than the nurses do.

‘The counselling starts with the counsellor; I think it’s most probably because they are more trained than us. We were all told somehow to do the counselling, the adherence counselling.’ (Participant 203, female, PN)

‘It (indicating adherence counselling) wasn’t taught during my nursing training.’ (Participant CHC 303, male, PN)

Nurses do, however, collaborate with external staff who are well trained to offer adherence counselling to PLHIV co-infected with TB.

‘Then maybe if let’s say here we use Non-Governmental Organisation for HIV/AIDS Care (ANOVA) with different companies for adherence counsellors; ANOVA and LIFELINE. So most of the time ANOVA does not have the adherence counsellors that are trained for adherence. So then LIFELINE has got the adherence counsellors.’ (Participant CHC401, female, PN)

The above quotes allude to the fact that these nurses seldom carry out adherence counselling, although it is perceived as important. This is largely because adherence counselling is not taught at the nursing training and LIFELINE has trained adherence counsellors specifically.

#### Category 1.3: Referral role as function of the nurses

The second role that emerged was the referral of HIV patients co-infected with TB to the HIV and TB counsellors at the CHCs as can be seen by these statements:

‘Then for the harder ones [indicating defaulters of more than 4 months] adherence counselling session, you refer them to the adherence counsellors.’ (Participant CHC101, female, EN)

‘We get a lot of patients that are defaulting. So, of course, then first thing that we do, we send them for adherence counselling. But fortunately for us, we do have counsellors that are doing tough, not all counsellors have got adherence counselling.’ (Participant CHC401, female, PN)

The above quotes indicate that PLHIV co-infected with TB that have defaulted their treatment for more than 4 months are referred to the adherence counsellors for adherence counselling. Also, from the observational point of view of this participant, many patients are defaulting their treatment plan. Fortunately for this CHC, they have trained adherence counsellors onsite.

### Theme 2: Display of professionalism by these nurses despite challenges

Creating a good nurse–patient relationship has an interpersonal impact when caring for PLHIV co-infected with TB. The nurses displayed professionalism and good human relations with PLHIV co-infected with TB despite their co-infection, substance use challenges,was perceived to be a significant theme.

#### Category 2.1: Importance of positive relations (encouragement and reassurance)

Participants reported that they created a friendly and conducive environment for the patients to win them back onto their treatment plan.

‘You must give a smile everyday so that even if he’s at home, he remembers your smile and come back for treatment…’ CHC202)

‘You have to sympathize with them. Because if you are not doing that, you will not know the reason why she is not taking the tablets if you are shouting. Instead, and you learn to listen to them…’ (Participant CHC202, male, EN)

‘Just be a person, not a nurse when you’re talking to them.’ (Participant CHC301, male, PN)

For the question, ‘Sister, do you think you have good adherence counselling skills to counsel this group of people that are infected with both HIV and TB?’, one participant said:

‘Yes, because you tell them; you must not be scared of these tablets, especially the youngsters. Some of them, they have co-infections, and another thing is alcohol and tobacco.’ (Participant CHC202, male, EN)

Professionalism through good human relations was also reported by a participant who felt that part of the adherence counselling skill was emphasising and repeating everything over again.

‘I think you try to emphasise and repeat everything over again. But the main thing about relapse is you find out the reasons for the relapse. But you must create that patient-nursing relationship so that the patient can trust you enough to disclose where the problem is lying…’ (Participant CHC203, female, PN)

In this theme, the quotes allude to the fact that these nurses show empathy and treatment adherence enforcement towards these patients.

### Theme 3: The role of expertise and experience

Adherence counselling from experience when they encountered clients who were not adhering to their treatment plan emerged as a strong theme. Their adherence counselling skill was based on their day-to-day interactions with people living with HIV co-infected with TB, as formal adherence counselling training was not done. Adherence counselling from experience at the points of treatment initiation, continuation and end of treatment did boost the adherence counselling skill of the nurses.

#### Category 3.1: Counselling from experience

Nine out of 14 participants who reported counselling from experience, however, felt that this was not an adequate adherence counselling service offered to PLHIV co-infected with TB at the point of treatment initiation, continuation and end of treatment. In responding to the question on what adherence counselling services they had been offering and how they acquired the skills, the following responses were received:

‘I didn’t get any formal training. It’s from working experience. No one trained me to counsel, I just counsel the patients as they present.’ (Participant CHC103, female, PN)

‘Day-to-day running of the TB and HIV patients, the experience of how we encounter with the patients is an individual thing here at the clinic.’ (Participant CHC203, female, PN)

‘It’s out of my own experience….’ (Participant CHC401, female, PN)

‘Out of experience. Because adherence counsellors do the counselling. So, but with the PACK TB/HIV guidelines, each and every time when the patient is coming you need to talk, to reinforce the adherence.’ (Participant CHC301, male, PN)

As reinforcement to treatment adherence should be ongoing at every clinic visit, and nurses provide this using their own experience, they should be trained on how to carry out adherence counselling.

### Theme 4: Perceived patient barriers to attend CHC follow-up appointments impacting on nurse’s role

A fourth theme emerged as the perception of the barriers which prevent patients from coming to the clinic, such as employment challenges and the community habit of sharing medication.

#### Category 4.1: Employer-related barriers

Most of the participants reported that some patients relapsed because of employment challenges that they had to manage.

‘They can’t get time off work. Their bosses don’t give them time to come to the clinic, you have to work with the patient to see if it’s comfortable they can come on their off days when they’re not at work so that it doesn’t clash with the work environment…’ (Participant CHC203, female, PN)

‘I was defaulting because I didn’t disclose to my employer and my employer doesn’t approve that I must come to the clinic all the time…’ (Participant CHC303, male, PN)

The lack of disclosure and employer support to attend their follow-up appointments at the CHCs greatly contribute to the non-adherence rate and the complexity of the work done by these nurses.

#### Category 4.2: Community-related barriers

A participant also reported on sharing of HIV and TB medications in the communities, hence not seeing the need to attend follow-up appointments at the CHCs.

‘No, sister, I didn’t, I defaulted according to you, but I was taking the tablets from my brother, from my wife, from my husband. They are sharing; I was getting the pills from my friend; my friend is getting a lot from his or her clinic so I was getting, so I was taking the tablets from her, so I didn’t default.’ (Participant CHC303, male, PN)

#### Category 4.3: Substance abuse

People living with HIV co-infected with TB were involved in heavy consumption of alcohol and heavy smoking whilst on treatment in the communities; some of the participants articulated this.

‘If you tell them another thing is alcohol; there’s tobacco. These people were drinking alcohol. Even before you see [attend to] them they were smoking. They can’t just cut abruptly right now. So you try to speak with them; you must try to cut. If it was 20 cigarettes, now they must come to 10. From 10 to 9, 8, 7, slowly, wean them. Because it’s not like, stop there now, no, stop today. And you tell them; you can’t just stop now but you must try to cut down because this is dangerous to your health. So they do understand; if you speak with them gentle and nice, they understand. Because these are the men of their houses and women of their house. Still you must respect them.’ (Participant CHC202, male, EN)

## Discussion

### The complexity of the varied roles of the nurses

#### Educational role

The study aimed to explore the perceptions of nurses regarding their adherence counselling skills of PLHIV co-infected with TB. The first major theme that emerged was the complexity of the varied roles of these nurses. The first role was the educational role of offering adherence counselling to PLHIV co-infected with TB at the CHCs. During the educational sessions with these patients, the nurses tried to educate them on the importance of taking their treatment every day and the fact that the treatment was a lifelong one. This was conducted through reassurance of the patients during the screening and diagnosing phase, before commencing treatment and during the treatment phase. Also emphasis on treatment follow up at the clinic. The educational role of the nurses is also responsible for reflecting on why treatment plans of some of these patients seem not to be working, supporting the view that adherence counselling is important to be included in their treatment plan (WHO 2007).

#### Counselling role

While TB and HIV-related counselling are predominantly provided by adherence counsellors who are trained to perform this task at the primary health care facilities (End Inequalities. End AIDS. Global AIDS Strategy 2021–2026), nurses are responsible for initiating patients on either ART or TB treatment (Sharma 2015). This study supports previous research (Mntlangula, Khuzwayo & Taylor 2017) that links to adherence counselling services for PLHIV co-infected with TB as part of the care role offered by the nurses. Counselling is working with people over a long or short period to help them bring effective change or enhance their well-being (Jenkins 2017). Participants reported being willing to offer adherence counselling based on their day-to-day experience with these patients, but implementation was lacking because of the challenges experienced by the nurses, such as having many patients to attend to on a daily basis, small consultation room sizes and the lack of in-service training on adherence counselling. This is supported by a study in a Burundi health care centre in Dar es Salam in Tanzania, where the nurses described their willingness to educate patients with co-infection and that adherence counselling of the patients is an important instrument in caring (Nilsson & Bölenius n.d.). However, challenges in terms of time and workload can result in a negative impact as adherence counselling services are not carried out, as shown in studies from Tanzania and United Kingdom (Havaei & MacPhee 2020; Nilsson & Bölenius 2015).

The counselling role is influenced by the collaboration with the counsellors who have been specifically trained to offer adherence counselling (Fernando & Msf 2014). This collaboration with support staff (adherence counsellors) sometimes posed a challenge in that often not all the counsellors working at the CHC were trained on adherence counselling, especially the ANOVA counsellors. ANOVA is an HIV and TB non-governmental organisation (NGO) that does not have trained HIV with TB adherence counsellors. Furthermore, the study revealed that the nurses were not actively involved in-depth adherence counseling. In most cases patients are referred to LIFELINE counselors who are trained adherence counselors and a Non Governmental Organization (NGO) (Motsoaledi 2016).

However, in this study, the majority of the participants reported that they felt that they did not possess adequate adherence counselling skills. Nurses are seen to be at the frontline in the health care sector, especially at the CHCs. Most of the interaction between the patients was noticed to take place between the nurse and these patients in the CHCs. In this study, the concept of adherence counselling of PLHIV co-infected with TB was noted to be lacking as the counselling skill required by the nurses to offer this service was inadequate (Jenkins 2017; Havaei & MacPhee 2020; Mntlangula et al. 2017). This lack of adequate adherence counselling skills in seven out of the 14 participants was noted as a gap. It should be noted that the South African National Department of Health compiled a comprehensive adherence guidelines document for HIV, TB and NCDs with strategies and procedures to improve linkage, adherence and retention in care (Dlamini 2016). These guidelines place adherence counselling as a core to remove the barriers to adherence. Yet implementation thereof has not been adequate, as seen in the analysis of this study.

The educational role of nurses does however serve as a source of acquiring skills to carry out adherence counselling for PLHIV co-infected with TB due to the close interactions of the nurses with these patients based on a positive patient–nurse relationship (Nilsson & Bölenius n.d.). This has been reported in the literature with a study in Addis Ababa on adherence counselling offered to a group of patients as one of the methods to help these patients overcome their psychological barriers to treatment follow-up and to encourage them develop self-treatment efficacy (Tola et al. 2016). This counselling intervention was provided by trained health care professionals at each TB and HIV clinic, once a week for the first month until the end of TB treatment (Tola et al. 2016). Adherence counselling intervention resulted in the decline of non-adherence levels significantly at the endpoint of the follow-up period (Tola et al. 2016). The present study supported this by identifying adherence-counselling services offered to PLHIV co-infected with TB as not new, as some of the nurses have been offering this service through education (Mntlangula et al. 2017). The use of reflection in adherence counselling is recommendable.

### Display of professionalism by these nurses despite challenges

The second theme that emerged was the professionalism of the nurses despite the ongoing challenges experienced. The study found that the nurses still upheld nursing ethics and displayed professionalism despite the challenge of inadequate adherence counselling skills. The term professionalism is closely related to the nursing profession, and providing professional nursing care is important for quality assurance in health care (Marcinowicz et al. 2020). Central to professionalism is the need for positive human relationships with encouragement and support, which were reported as being part of their role regardless of the challenges. Good nurse–patient relationship in other research was demonstrated by the majority of the participants who regarded adherence counselling of PLHIV co-infected with TB as important (Enane et al. 2020; Nilsson & Bölenius n.d.). Most of the nurses created a friendly and conducive environment for the patients to win them back onto their treatment plan; this concurred with a study in Tanzania (Nilsson & Bölenius n.d.). Some of the nurses emphasised that defaulters should not be admonished, as most of them have serious family and personal issues.

### The role of expertise and experience

The third theme which emerged was the role of expertise and experience. Some of the nurses felt they did not have adequate adherence counselling skills at the point of treatment initiation, continuation and end of treatment. However, they were able to counsel these patients from their experience in their role. Their adherence counseling was based on their experience caring for PLHIV co-infected with TB and background from nursing training as students. Future research needs to explore the challenges encountered by nurses when offering adherence counselling services to these patients.

### Perceived barriers to attend CHC follow-up appointments

The final theme that emerged was the perceived barriers that patients experienced in attending CHS and the impact this can have on their role. These issues were complex and included medication-related factors, alcohol abuse and fear of losing their jobs (Bauleth et al. 2017). Three barriers were identified in this study. A major barrier is work-related issues. These employer-related challenges were like the challenges reported by the patients in a study in Windhoek District, Namibia. A second barrier was the community practice of sharing of ARVs and TB tablets in the communities as reported by the participants. This finding correlated with the reports from Embon in KwaZulu-Natal, where these patients were sharing these chronic medications among themselves (Chibi, Yende-Zuma & Mashamba-Thompson 2020). The health workers explained to the community that this could lead to early depletion of ARV stock issued to a patient, putting their life at risk, increased drug resistance and ultimately failing the medication regimen (South Africa health E-NEWS 2019).

## Conclusion

In conclusion, most of the participants highlighted that their role was complex and included education and counselling, but they did not have adequate adherence counselling skills because they have not received adherence counselling training. However, they could offer adherence counselling services to PLHIV co-infected with TB through their roles of educating, counselling from experience and referral to the HIV and TB counsellors for deep counselling. Despite the lack of counselling skills, the majority of the participants still maintained work professionalism and showed empathy towards the patients.

## Limitations of the study

A major limitation of this study was that nurses were not only providing counselling, but also other services, and more time with the patient is required. This was due to the huge number of patients to be attended to. Future research could seek into the Key Performance Areas (KPA) of these nurses. Also, this study was conducted only in one health sub-district in Cape Town.

Moreover, this study was conducted in the Khayelitsha health sub-district only. This was because the Khayelitsha health sub-district was purposively selected, as they service the highest number of PLHIV and TB based on head count and recorded the highest numbers of loss-to-follow-up of PLHIV co-infected with TB. Future research could conduct a comparative study with other Cape Town health sub-districts like the Northern Panorama health sub-district.
